# Biomarkers and Algorithms for the Diagnosis of Vitamin B_12_ Deficiency

**DOI:** 10.3389/fmolb.2016.00027

**Published:** 2016-06-27

**Authors:** Luciana Hannibal, Vegard Lysne, Anne-Lise Bjørke-Monsen, Sidney Behringer, Sarah C. Grünert, Ute Spiekerkoetter, Donald W. Jacobsen, Henk J. Blom

**Affiliations:** ^1^Laboratory of Clinical Biochemistry and Metabolism, Department for Pediatrics, Medical Center, University of FreiburgFreiburg, Germany; ^2^Department of Clinical Sciences, University of BergenBergen, Norway; ^3^Department of Clinical Biochemistry, Haukeland University HospitalBergen, Norway; ^4^Department of Cellular and Molecular Medicine, Lerner Research Institute, Cleveland ClinicCleveland, OH, USA

**Keywords:** vitamin B_12_, cobalamin, homocysteine, methylmalonic acid, holo-transcobalamin, diagnostic algorithm, functional deficiency of B_12_

## Abstract

Vitamin B_12_ (cobalamin, Cbl, B_12_) is an indispensable water-soluble micronutrient that serves as a coenzyme for cytosolic methionine synthase (MS) and mitochondrial methylmalonyl-CoA mutase (MCM). Deficiency of Cbl, whether nutritional or due to inborn errors of Cbl metabolism, inactivate MS and MCM leading to the accumulation of homocysteine (Hcy) and methylmalonic acid (MMA), respectively. In conjunction with total B_12_ and its bioactive protein-bound form, holo-transcobalamin (holo-TC), Hcy, and MMA are the preferred serum biomarkers utilized to determine B_12_ status. Clinically, vitamin B_12_ deficiency leads to neurological deterioration and megaloblastic anemia, and, if left untreated, to death. Subclinical vitamin B_12_ deficiency (usually defined as a total serum B_12_ of <200 pmol/L) presents asymptomatically or with rather subtle generic symptoms that oftentimes are mistakenly ascribed to unrelated disorders. Numerous studies have now established that serum vitamin B_12_ has limited diagnostic value as a stand-alone marker. Low serum levels of vitamin B_12_ not always represent deficiency, and likewise, severe functional deficiency of the micronutrient has been documented in the presence of normal and even high levels of serum vitamin B_12_. This review discusses the usefulness and limitations of current biomarkers of B_12_ status in newborn screening, infant and adult diagnostics, the algorithms utilized to diagnose B_12_ deficiency and unusual findings of vitamin B_12_ status in various human disorders.

## Vitamin B_12_ deficiency

**Figure 1:**
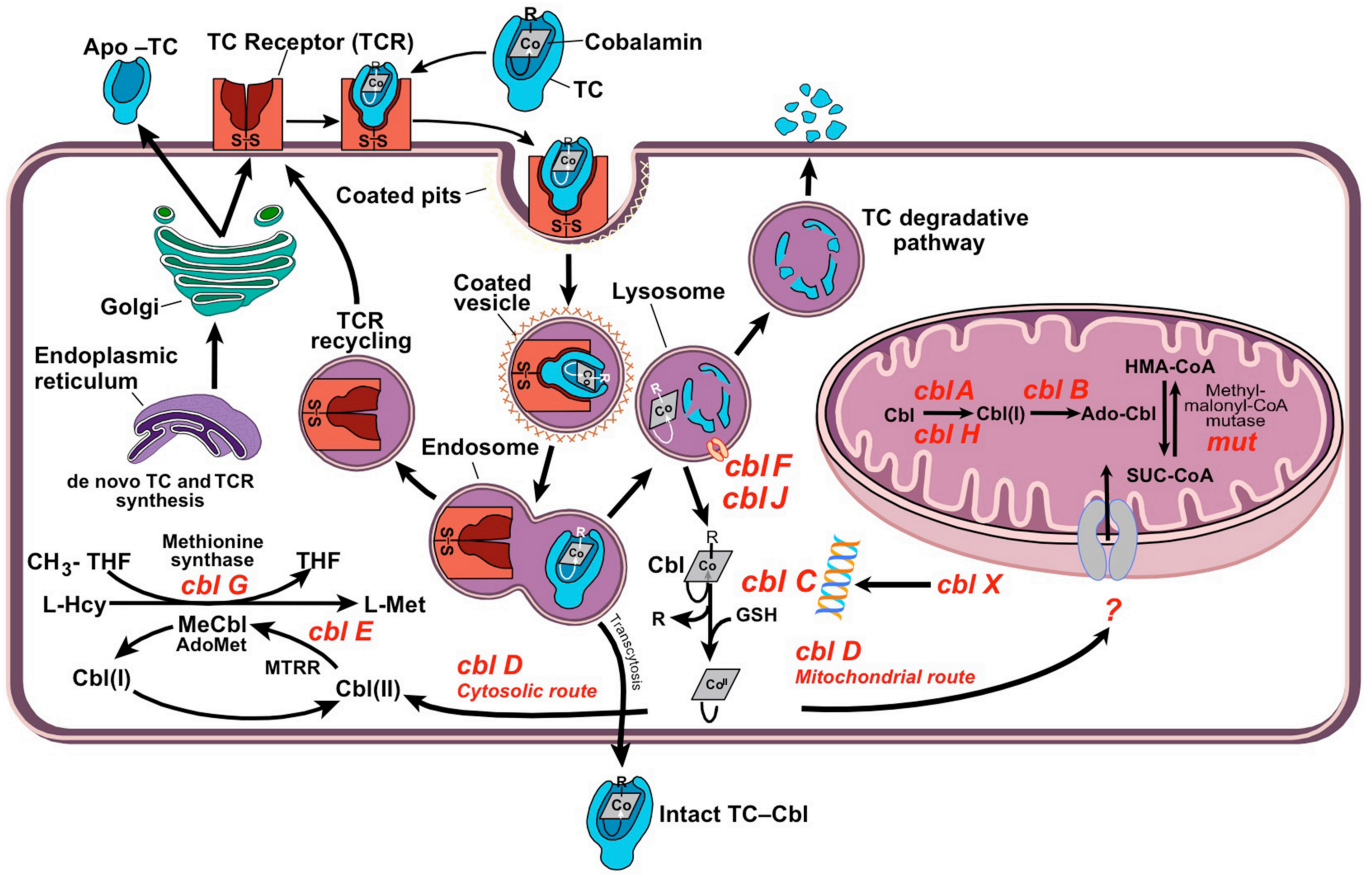
**Cellular processing and trafficking of dietary vitamin B_12_**. Cells take up holo-TC via TC-receptor-mediated endocytosis (TCR). In the lysosome, cobalamin (Cbl) is liberated and apo-transcobalamin is degraded, while the TCR is recycled back to the cell surface. Cbl exits the lysosome via transporters cblF/cblJ. In the cytosol, Cbl undergoes processing by enzyme cblC, which catalyzes removal of the upper-axial ligand (R), reduction of the cobalt center and conversion into the base-off configuration. The fate of newly processed B_12_ is dictated by cblC-cblD interactions, which direct the cofactor into either cytosolic methionine synthase (MS, cblG) or mitochondrial methylmalonyl-CoA mutase (MCM, mut). Nutritional and functional deficiencies of B_12_ lead to the inactivation of client enzymes MS and MCM with elevations of tHcy and MMA.

Vitamin B_12_ (B_12_ = Cbl, “cobalamin,” the chemical name) is an essential water-soluble micronutrient required by all cells in the body. Humans are unable to synthesize B_12_ and thus rely on dietary intakes and a complex intracellular route for vitamin processing and delivery to its target destinations (Figure 1) (Hannibal et al., 2009). Vitamin B_12_ deficiency due to malabsorption and inadequate intake is a public health issue worldwide. It is estimated that 15–20% of the elderly in the United States are B_12_ deficient (Allen, 2009). In Germany, about 10% of the male elderly population and 26% of the female elderly population present with insufficient levels of vitamin B_12_ (Hartmann, 2008; Grober et al., 2013). In India, ~75% of the population, i.e., over 650 million people, have B_12_ deficiency (Antony, 2001; Refsum et al., 2001), which can only be partly ascribed to a vegetarian diet in a substantial portion of the population.

Vitamin B_12_ deficiency is a multifactorial condition caused by insufficient intake (nutritional deficiency) as well as acquired or inherited defects that disrupt B_12_ absorption, processing and trafficking pathways (functional deficiency). Methylcobalamin (MeCbl) serves as a coenzyme for the biosynthesis of methionine from homocysteine catalyzed by the cytosolic enzyme methionine synthase (MS). This reaction regenerates tetrahydrofolate (THF) from *N*^5^-methyl-tetrahydrofolate (*N*^5^-CH_3_-THF), which is essential for the *de novo* biosynthesis of nucleic acids. Adenosylcobalamin (AdoCbl) is required for the conversion of methylmalonyl-CoA to succinyl-CoA catalyzed by mitochondrial methylmalonyl-CoA synthase (MCM), an anaplerotic reaction that furnishes increased demands for the Krebs cycle and heme biosynthesis precursor succinyl-CoA.

Insufficient supply of B_12_ and genetic defects impairing its cellular processing and trafficking lead to the accumulation of homocysteine (Hcy) and methylmalonic acid (MMA), which enter circulation and give rise to hyperhomocystinemia and methylmalonic acidemia.

The recommended daily dose of B_12_ for adults is 2.4 μg per day (Institute of Medicine (US) Standing Committee on the Scientific Evaluation of Dietary Reference Intakes and its Panel on Folate, Other B Vitamins, and Choline, 1998), which is equivalent to the daily intake in the normal western diet. Malabsorption due to aging, poor nutrition and acquired defects in vitamin B_12_ metabolism are the leading causes of vitamin B_12_ deficiency worldwide. Inborn errors of vitamin B_12_ metabolism are rare.

Vitamin B_12_ deficiency is frequently under-diagnosed in pregnancy and in infants from mothers having insufficient levels of the micronutrient (Wheeler, 2008; Sarafoglou et al., 2011). Ensuring sufficient intake of vitamin B_12_ during pre-conception, pregnancy, and post-partum is strongly recommended (Bjørke Monsen et al., 2001; Rasmussen et al., 2001; Bjørke-Monsen et al., 2008; Hinton et al., 2010; Dayaldasani et al., 2014). Other populations at risk of developing vitamin B_12_ deficiency include the elderly, vegetarians and vegans, recipients of bariatric surgery (Majumder et al., 2013; Kwon et al., 2014) as well as those suffering from gastrointestinal diseases featuring ileal resections >20 cm (Battat et al., 2014). Certain medications such as metformin (Greibe et al., 2013b; Aroda et al., 2016) and proton-pump inhibitors (Howden, 2000; Wilhelm et al., 2013) may also transiently induce a status of cobalamin deficiency, which may be reversible upon completion of treatment and/or with oral vitamin B_12_ supplementation.

Herein, we discuss three aspects of the assessment of B_12_ status: (1) The utility of metabolites used as biomarkers of vitamin B_12_ deficiency in neonates and adults, (2) The algorithms employed to predict subclinical and clinical B_12_ deficiency, and (3) Major challenges and diagnosis of vitamin B_12_ deficiency in special populations.

## Serum biomarkers of vitamin B_12_ deficiency: strengths and limitations

### Total serum vitamin B_12_

The most direct assessment and perhaps preferred first-assay to determine vitamin B_12_ status is the measurement of total serum vitamin B_12_. This assay is widely available in clinical chemistry laboratories. Ranges for normal (>250 pmol/L), low (150–249 pmol/L), and acute deficiency (<149 pmol/L) vitamin B_12_ have been defined and are used in most clinical chemistry laboratories worldwide (Clarke et al., 2003; Selhub et al., 2008; Mirkazemi et al., 2012). One limitation of this biomarker is that it assesses total circulating vitamin B_12_, of which ~80% is bound to haptocorrin, and therefore, not bioavailable for cellular uptake. Another limitation of this assay lies in its unreliability to reflect cellular vitamin B_12_ status. Results from studies assessing serum and cellular vitamin B_12_ have shown that the levels of serum B_12_ do not always represent cellular B_12_ status (Carmel, 2000; Solomon, 2005; Devalia et al., 2014; Lysne et al., 2016). In particular, patients with inborn errors of vitamin B_12_ metabolism can present with normal or low serum values of the vitamin, while being deficient at the cellular level. Furthermore, functional vitamin B_12_ deficiency due to oxidative stress has been identified in elders exhibiting normal values of serum vitamin B_12_ (Solomon, 2015). Functional deficiency of vitamin B_12_ was corrected upon supplementation with cyanocobalamin (CNCbl), as judged by reduction in the serum levels of tHcy and MMA (Solomon, 2015). Thus, total serum B_12_ is not a reliable biomarker of vitamin B_12_ status when used alone. Nonetheless, this marker should not be considered obsolete as a number of studies show that total serum vitamin B_12_ may be helpful to predict prognosis and status of diseases featuring abnormally high vitamin B_12_ levels in serum (>650 pmol/L), such as cancer (Arendt et al., 2016) and autoimmune lymphoproliferative syndrome (ALPS) (Bowen et al., 2012).

### Homocysteine

**Figure 2:**
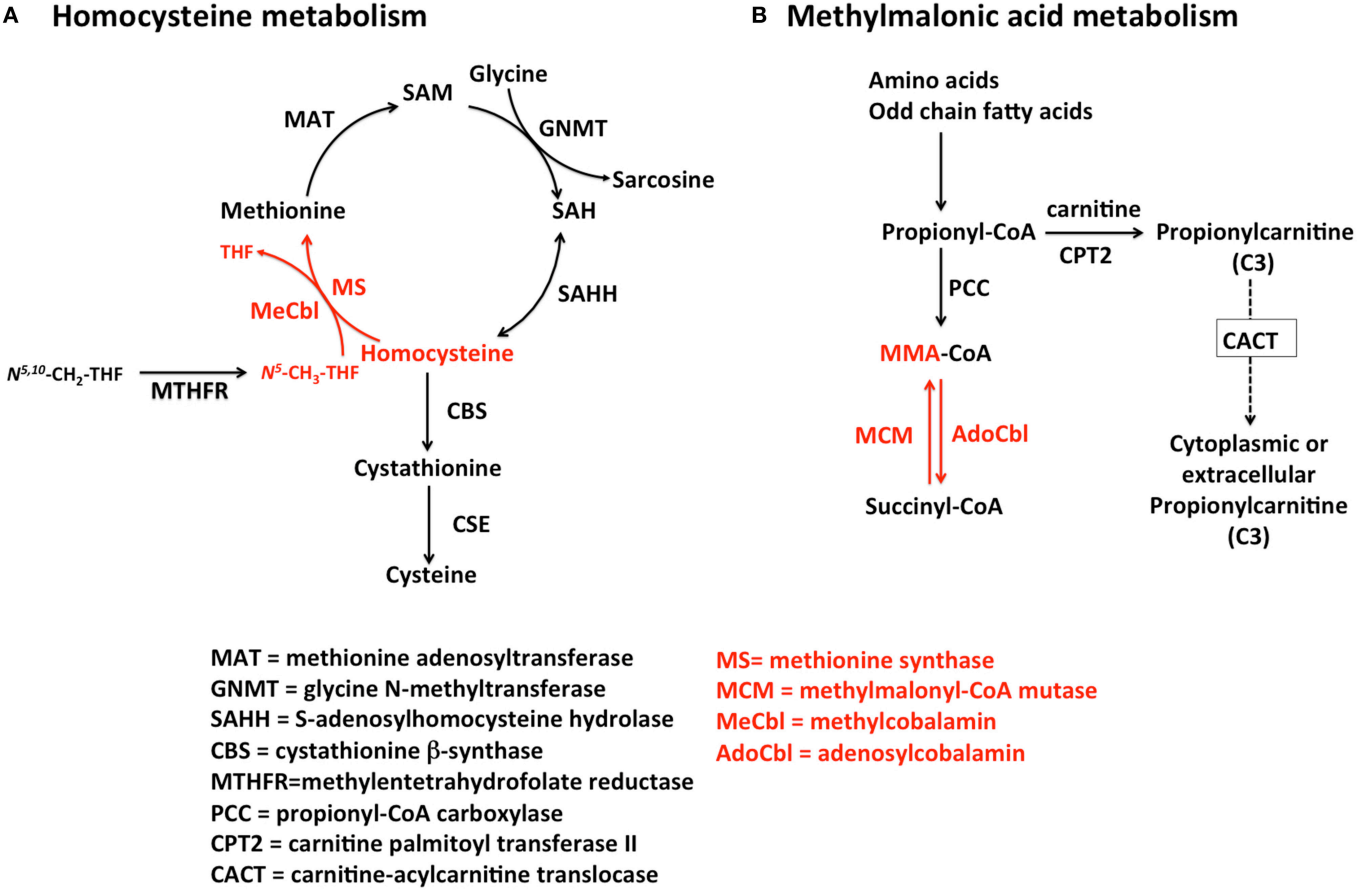
**Pathways for Hcy and MMA metabolism in humans**. **(A)** Homocysteine is a branch-point metabolite at the intersection of either the remethylation or the transsulfuration pathways. Thus, Hcy homeostasis relies on three different biochemical reactions [MS, cystathionine β-synthase (CBS) and S-adenosylhomocysteine hydrolase (SAHH)], two of which (CBS and SAHH) are independent of vitamin B_12_. In addition to nutritional deficiency of vitamin B_12_, elevation of Hcy in plasma may arise from reduced function of CBS and MTHFR, as well as nutritional deficiencies of folate. **(B)** MMA is produced during catabolism of odd-chain fatty acids and amino acids in the mitochondrion. Propionyl-CoA is the precursor of MMA in a reaction catalyzed by propionyl-CoA carboxylase (PCC). Inborn errors of PCC lead to propionic acidemia. Likewise, mutations in AdoCbl-dependent MCM lead to a buildup of MMA-CoA and inhibition of PCC that manifests as increased propionyl-CoA and so of propionic acid the circulation. Propionylcarnitine can also be transported out of the cell to reach systemic circulation. Propionylcarnitine is a first-line test in newborn screening.

Homocysteine is a metabolite of one-carbon metabolism that is remethylated by MeCbl-dependent MS or betaine-homocysteine methyltransferase as part of the methionine cycle (Finkelstein and Martin, 1984) and degraded by cystathionine β-synthase (CBS) in the transsulfuration pathway (Figure 2). Conversion of Hcy to Met by MS depends on the availability of both vitamin B_12_ and folate (as *N*^5^-CH_3_-THF), and therefore, nutritional deficiencies in either one of these micronutrients lead to the accumulation of Hcy in serum and urine. Likewise, inborn errors of metabolism that impair the upstream processing and trafficking of B_12_ or folate lead to elevation of this metabolite, a condition collectively known as hyperhomocystinemia. The normal range of total plasma Hcy (tHcy) in human plasma is 5–15 μmol/L (Ueland et al., 1993) and values >13 μmol/L may be considered elevated in adults (Jacques et al., 1999). Homocysteine levels are always higher in serum compared to plasma due to the release of Hcy bound to cellular components (Jacobsen et al., 1994). Hence, plasma and not serum should be used to determine the levels of tHcy. Although gender and age reference intervals have been established in some studies (Jacobsen et al., 1994; Rasmussen et al., 1996; van Beynum et al., 2005), they are usually ignored in the reporting of tHcy levels. Because of the dual biochemical origin of elevated Hcy, this biomarker is of limited value to assess vitamin B_12_ status as a stand-alone measurement. This is also true for the newborn screening of inborn errors of vitamin B_12_ metabolism found in the cblD, cblF, and cblJ (Huemer et al., 2015) disorders.

### Methylmalonic acid

MMA increases upon inactivation of AdoCbl-dependent MCM in the mitochondrion. Nutritional and functional deficiencies of vitamin B_12_ result in the inactivation of MCM leading to buildup of its substrate methylmalonyl-CoA, which enters circulation as free MMA. The reaction catalyzed by MCM (Figure 2) is not affected by other vitamins of one-carbon metabolism, and therefore, MMA is considered a more specific marker of vitamin B_12_ deficiency (Clarke et al., 2003). Serum values of MMA, ranging from >260 to 350 nmol/L indicate elevation of this metabolite (Clarke et al., 2003). Nonetheless, there are few pathologies such as renal insufficiency that lead to an increase in MMA (Iqbal et al., 2013). For example, one study showed that 15–30% of individuals with high vitamin B_12_ concentrations in serum also had elevated MMA concentrations, which may reflect renal dysfunction instead of authentic vitamin B_12_ deficiency (Clarke et al., 2003). Thus, the utility of this marker should be considered carefully in older patients and patients with suspected or established renal disease. Assessment of a second marker of vitamin B_12_ status, such as holo-transcobalamin (holo-TC) (Iqbal et al., 2013) should be considered. Another study showed that the clearance of both Hcy and MMA may be compromised in patients with reduced kidney function (Lewerin et al., 2007).

### Total serum holo-transcobalamin

Dietary B_12_ is transported in the digestive system via the use of three protein transporters that bind the micronutrient in a sequential fashion, following the order haptocorrin (HC), intrinsic factor (IF), and transcobalamin (TC) (Fedosov et al., 2007; Fedosov, 2012). After absorption in the intestine, vitamin B_12_ bound to TC (holo-TC) reaches circulation and it is distributed to every cell in the body. Cells take up holo-TC via receptor-mediated endocytosis, aided by the transcobalamin receptor (TCblR; CD320) (Quadros et al., 2009). Because the only fraction of dietary vitamin B_12_ that is bioavailable for systemic distribution is in the form of holo-TC (Valente et al., 2011), the level of holo-TC in serum has been successfully utilized as a marker of bioactive vitamin B_12_ (Nexo et al., 2000, 2002; Valente et al., 2011; Yetley et al., 2011). Holo-TC represents 6–20% of the total vitamin B_12_ present in serum (Nexo et al., 2000, 2002; Valente et al., 2011; Yetley et al., 2011). This marker is more accurate in assessing the biologically active fraction of vitamin B_12_ in circulation than serum B_12_ itself, and its level correlates well with the concentration of vitamin B_12_ in erythrocytes (Valente et al., 2011). The diagnostic value of holo-TC has proven superior to Hcy and MMA for the assessment of vitamin B_12_ status in the elderly (Valente et al., 2011). The normal range of holo-TC in healthy subjects is 20–125 pmol/L (Valente et al., 2011). Additional research is needed to elucidate the mechanisms that control holo-TC homeostasis in the normal population and in pathologies that alter vitamin B_12_ transport and utilization. For example, abnormally low levels of holo-TC have been documented in patients receiving chemotherapy, macrocytosis and in individuals carrying the TC polymporphism 67A>G, without vitamin B_12_ deficiency (Vu et al., 1993; Wickramasinghe and Ratnayaka, 1996; Riedel et al., 2005, 2011). Insufficient sensitivity (44%) of holo-TC as a marker of vitamin B_12_ status was noted in a cohort of 218 institutionalized elderly patients (Palacios et al., 2013). At present time, it is unknown whether and how holo-TC levels vary in patients harboring inborn errors affecting intracellular vitamin B_12_ metabolism (cblA-cblJ). Thus, the diagnostic value of holo-TC as a first line test awaits further investigation.

### Propionylcarnitine (C3) and its ratios with acetylcarnitine (C3/C2) and palmitoylcarnitine (C3/C16)

In addition to the four canonical markers discussed above, vitamin B_12_ deficiency leads to accumulation of propionylcarnitine (C3) (Sarafoglou et al., 2011), which is a marker of methylmalonic aciduria and propionic acidemia. While vitamin B_12_ deficiency is not a primary test in newborn screening programs, a study showed that markers C3, C3/C2, and C3/C16 exhibit a negative correlation with maternal levels of vitamin B_12_ in the first trimester of pregnancy, and thus may bear diagnostic value (Dayaldasani et al., 2014). Although this finding requires further investigation with respect to the functional biomarkers Hcy and MMA, the finding opens the possibility of early identification of vitamin B_12_ insufficiency or deficiency during the first trimester of pregnancy and adequate treatment.

## Algorithms for the diagnosis of vitamin B_12_ deficiency

According to the World Health Organization (WHO), vitamin B_12_ status in adults is defined by the serum levels of the micronutrient with the following cut-offs and definitions: >221 pmol/L is vitamin “B_12_ adequacy”; between 148 and 221 pmol/L is “low B_12_,” and lower than 148 pmol/L is “B_12_ deficiency” (de Benoist, 2008; Allen, 2009). However, stand-alone markers of B_12_ status, such as serum B_12_, have proven insufficient for the unequivocal diagnosis of vitamin B_12_ deficiency (Fedosov, 2013; Palacios et al., 2013; Remacha et al., 2014; Fedosov et al., 2015). Further, the WHO criterion does not account for age effects. Algorithms that combine a minimum of two biomarkers have been employed worldwide, each exhibiting advantages and disadvantages (Palacios et al., 2013; Remacha et al., 2014).

A study performed on a Swedish population proposed that when physicians request testing for suspected vitamin B_12_ or folate deficiency, the first-line test of choice should be tHcy, and only when tHcy > 9 μM, should additional markers be tested to discriminate between vitamin B_12_ and folate deficiencies (Schedvin et al., 2005). This approach proved effective in reducing diagnostic costs by 30% (Schedvin et al., 2005).

Herrmann and Obeid proposed a two-step algorithm for the diagnosis of vitamin B_12_ in adults that utilizes holo-TC and MMA as biomarkers (Herrmann and Obeid, 2013). Analysis of 1359 samples submitted to the laboratory for total vitamin B_12_ assessment showed that patients exhibiting holo-TC values between 23 and 75 pmol/L and normal renal function should also be tested for MMA (Herrmann and Obeid, 2013). This guideline was widely recommended in Germany (Herrmann and Obeid, 2008) for the diagnosis of vitamin B_12_ deficiency in risk groups including infants, unexplained anemia, unexplained neuropsychiatric symptoms, gastrointestinal conditions including stomatitis, anorexia and diarrhea, the elderly, vegetarians and individuals with gastrointestinal diseases such as ilium resection, chronic atrophic gastritis, Crohn's disease and *Helicobacter pylori* infection and individuals under treatment with proton-pump inhibitors (Herrmann and Obeid, 2008; Hartmann et al., 2009; Koch, 2009; Heinzl, 2014). A cutoff of holo-TC of 50 pmol/L was set to discriminate between individuals unlikely to have vitamin B_12_ deficiency (holo-TC > 50 pmol/L) vs. those potentially deficient in the micronutrient (holo-TC < 50 pmol/L) (Herrmann and Obeid, 2008). Patients with potential vitamin B_12_ deficiency were first stratified by holo-TC levels as very low (holo-TC < 35 pmol/L) and low (holo-TC 36–50 pmol/L), and a second-line testing of MMA follows thereafter. Results from MMA lead to a three-block classification of patients, where (a) MMA < 271 nmol/L with holo-TC < 35 pmol/L represents a negative vitamin B_12_ balance (insufficiency), (b) MMA < 271 nmol/L with holo-TC 36–50 pmol/L suggests the patient is unlikely to be vitamin B_12_ deficient, and (c) a range of possible vitamin B_12_deficient patients is characterized by MMA > 271 nmol/L with holo-TC being very low or low (Herrmann and Obeid, 2008).

Berg and Shaw presented a cascade-testing algorithm that stratified patients first by total serum vitamin B_12_ levels and second by MMA levels, using cutoffs of 118 pmol/L and 0.80 μmol/L, respectively (Berg and Shaw, 2013). The authors encouraged the sequential measurement of these two biomarkers prior to the implementation of vitamin B_12_ therapy in patients.

Guidelines from the British Committee for Standards in Hematology suggested the use of total serum vitamin B_12_ as the first-line test, with MMA as the second-line test (Devalia et al., 2014). For the reasons presented in the sections above, this approach would exclude a significant fraction of patients for which serum vitamin B_12_ does not reflect cellular, genetic, and pharmacological disturbances that lead to functional vitamin B_12_ deficiency. It was also recommended that individuals classified as having “subclinical deficiency” be provided with empirical therapy with oral CNCbl 50 μg daily for 4 weeks, and have their serum B_12_ levels re-checked after 3 months (Devalia et al., 2014). Immediate medical attention was recommended for patients with symptoms of neuropathy.

Studies conducted by Palacios et al. on a cohort of 218 institutionalized elderly patients with median age 80 years old showed that an algorithm that combined biochemical, hematological, and morphological data proved more effective for the diagnosis of vitamin B_12_ than the isolated markers (Palacios et al., 2013). The proposed algorithm combines erythrocyte and serum folate, holo-TC, and MMA, and excludes serum vitamin B_12_ and tHcy measurements. The biomarkers selected in this algorithm permit the discrimination between isolated folate and vitamin B_12_ deficiencies as well as the combined deficiency of both vitamins (Palacios et al., 2013).

Fedosov and colleagues developed a series of equations that combine two, three, or four biomarkers of vitamin B_12_ status in adults. Age effects and folate status, a modifier of vitamin B_12_ metabolism, are also considered (Fedosov et al., 2015). The combined indicator of vitamin B_12_ status, cB_12_, is defined as: cB_12_ = log10((holo-TC.B_12_)/(MMA.tHcy))−(age factor) (Fedosov et al., 2015), and thus, it differs from the “if → then” structure of classic diagnostic algorithms. The combined indicator of vitamin B_12_ status provides five distinct ranges of diagnostic value, which are summarized in Table 1. This classification considers both extremes of vitamin B_12_ diagnostics, i.e., very low and very high vitamin B_12_ status. The pathophysiological implications of high vitamin B_12_ status are poorly understood, but its occurrence has been reviewed in the literature (Arendt and Nexo, 2013). The cB_12_ quotient has been successfully adopted to investigate the vitamin B_12_ status of a cohort of healthy Swiss elders (Risch et al., 2015). Results from this study suggest that increased levels of MMA and Hcy in seniors are brought about by a reduced renal function due to aging rather than by an underlying vitamin B_12_ insufficiency (Risch et al., 2015). Nonetheless, the study identified a clear trend of more prevalent metabolic vitamin B_12_ deficiency (measured as cB_12_) with increasing age (Risch et al., 2015). Another study showed that the cB_12_ indicator was useful not only to identify vitamin B_12_ deficiency, but also to find interactions with folate status (Brito et al., 2016). Further, the cB_12_ indicator was effective in identifying vitamin B_12_ in cancer patients who exhibit normal serum vitamin B_12_, but increased levels of MMA and Hcy (Vashi et al., 2016). In this study, the cB_12_ quotient helped to establish that MMA is the most sensitive marker of vitamin B_12_ in cancer patients, and that total serum B_12_ has very little diagnostic value (Vashi et al., 2016). None of the studies included a decrease in MMA or tHcy upon B_12_ administration as a potential marker in the evaluation of potential B_12_ deficiency.

**Table 1:** **Ranges of combined vitamin B_12_ status, their equivalence to single cut-off values, and clinical interpretation**.

**Combined vitamin B_12_ status**	**Equivalence to single cut-points**	**Interpretation**
Elevated B_12_ >1.5	B_12_ > 650 Holo-TC > 190 tHcy < 8.0 MMA < 0.11	The biological effects of high vitamin B_12_ are not fully understood
Adequate B_12_ −0.5 to 1.5	186 < B_12_ < 650 37 < holo-TC < 190 13.6 > tHcy > 8.0 0.35 < MMA < 0.11	Expected to support normal B_12_-dependent functions
Low B_12_ −1.5 to −0.5	119 < B_12_ < 186 20 < holo-TC < 37 19.2 > tHcy > 13.6 0.84 < MMA < 0.35	Subclinical deficiency. No hematological changes, subtle neurological impairment
Possible B_12_ deficiency −2.5 to −1.5	116 < B_12_ < 119 8.4 < holo-TC < 20 51 > tHcy > 19.2 1.7 < MMA < 0.84	Potential manifestations of vitamin B_12_ deficiency
Probable B_12_ deficiency < −2.5	B_12_ < 116 holo-TC < 8.4 tHcy > 51 MMA > 1.7	Clinical manifestations of vitamin B_12_ deficiency

Units: B_12_ and holo-TC are expressed in pmol/L, and tHcy and MMA in μmol/L. Table adapted from Fedosov et al. (2015).

## Vitamin B_12_ deficiency in infants

Infant cobalamin status at birth depends on maternal cobalamin stores during pregnancy, placental function, gestational age, and birth weight. There is a complex cobalamin metabolism in the placental-fetal compartment, strictly regulating the cobalamin transfer to the fetus (Porck et al., 1983; Miller et al., 1993; Perez-D'gregorio and Miller, 1998; Obeid et al., 2006). Cobalamin and holo-TC levels in the placenta, cord blood, and newborn serum correlate with maternal levels, but are 2–3 fold higher (Luhby et al., 1958; Baker et al., 1975; Giugliani et al., 1985; Frery et al., 1992; Obeid et al., 2006), providing the newborn with a liver store of about 25 μg cobalamin assumed to be sufficient for the first year of life (McPhee et al., 1988).

A wide range of cobalamin levels, varying from 86 to 939 pmol/L (median of 264 pmol/L) (Minet et al., 2000), have been reported for neonates during the first month of life. Maternal cobalamin deficiency, prematurity, and low birth weight are all associated with lower fetal cobalamin stores and an increased risk of deficiency, particularly if the infant is exclusively breastfed for more than 4 months before animal food is introduced (Dror and Allen, 2008; Torsvik et al., 2015). In mainly breastfed infants, the cobalamin level remains at cord blood levels for 5–7 days, then drops sharply, followed by a slow decline, reaching nadir levels at 6–7 months (Minet et al., 2000; Fokkema et al., 2002; Monsen et al., 2003). The decrease in serum cobalamin is accompanied by an increase in the metabolic markers plasma tHcy and MMA (Minet et al., 2000; Bjørke Monsen et al., 2001). This metabolic profile, indicative of cobalamin deficiency (Schneede et al., 1994), is associated with functional motor impairment in infants (Torsvik et al., 2013, 2015). Randomized intervention studies with one-time injection of 400 μg hydroxycobalamin are reported to improve the metabolic pattern and gross motor development compared with infants receiving placebo (Torsvik et al., 2013, 2015).

Reported cobalamin levels in human breast milk vary from 150 to 700 pmol/L (Specker et al., 1990a; Ford et al., 1996; Greibe et al., 2013a; Duggan et al., 2014), depending on the assay used (Black et al., 1994; Lildballe et al., 2009), maternal cobalamin levels and the stage of lactation, as cobalamin levels in breast milk falls progressively during the first months (Greibe et al., 2013a). The cobalamin content of breast milk appears to be lowest at 4 months after birth, and this coincides with a low cobalamin status in the infant (Greibe et al., 2013a). Gradually decreasing cobalamin status with increasing time of exclusively breastfeeding has also been reported in infants with a subnormal birthweight (2–3 kg) during the first 6 months of life (Torsvik et al., 2015). Most commercially prepared infant formulas are enriched with cobalamin up to concentrations of 800–1200 pmol/L, well above that of human milk (Ford et al., 1996), and higher cobalamin and lower tHcy and MMA levels are seen in formula-fed infants (Specker et al., 1990b; Minet et al., 2000; Fokkema et al., 2002; Hay et al., 2008).

After introduction of animal food, the cobalamin level increases and peaks at 3–7 years and then decreases, median plasma tHcy decreases and remains low until 7 years when it starts increasing, whereas median plasma MMA decreases after 12 months and remains low throughout childhood (Monsen et al., 2003). tHcy is considered the best metabolic marker of cobalamin status in infants and toddlers and a plasma tHcy cut-off level of 6.5 μmol/L has been suggested for defining cobalamin deficiency in this age-group (Bjørke-Monsen et al., 2008). This represents the 97.5 percentile in 4 months old infants given a single intramuscular dose of 400 μg hydroxocobalamin at 6 weeks, rendering them to be cobalamin optimized (Bjørke-Monsen et al., 2008). In addition, administration of folinic acid in newborns did not reduce tHcy (Hogeveen et al., 2010). In older children and adults, tHcy is mainly a folate marker, while MMA is considered a good marker for cobalamin status (Bjørke Monsen and Ueland, 2003; Monsen et al., 2003).

## Challenges in the diagnosis of vitamin B_12_ deficiency

### Subclinical B_12_ deficiency is asymptomatic

The diagnosis and management of subclinical vitamin B_12_ deficiency, which is defined as a total serum B_12_ concentration of 150–249 pmol/L(Carmel, 2012, 2013) is a matter of great interest, due to its much higher prevalence (up to 40% of the population in western countries) compared to clinical deficiency (Carmel, 2012, 2013). The etiology of subclinical vitamin B_12_ is unknown (Carmel, 2013). The condition manifests without overt clinical findings, with marginal or no elevation in tHcy and MMA and is typically of non-malabsorptive causes (Carmel, 2013). Subclinical B_12_ deficiency rarely evolves into clinical deficiency and the need for treatment with B_12_ has not been fully established in spite of its much higher frequency compared to clinical B_12_ deficiency (Carmel, 1996, 1998, 1999, 2011, 2013). A study with a population of asymptomatic B_12_-deficient elderly Chileans showed that patients displayed improved function of myelinated peripheral nerves after vitamin B_12_ treatment and a positive association with folate status (Brito et al., 2016).

### Functional B_12_ deficiency: serum markers vs. cellular status of vitamin B_12_

**Figure 3:**
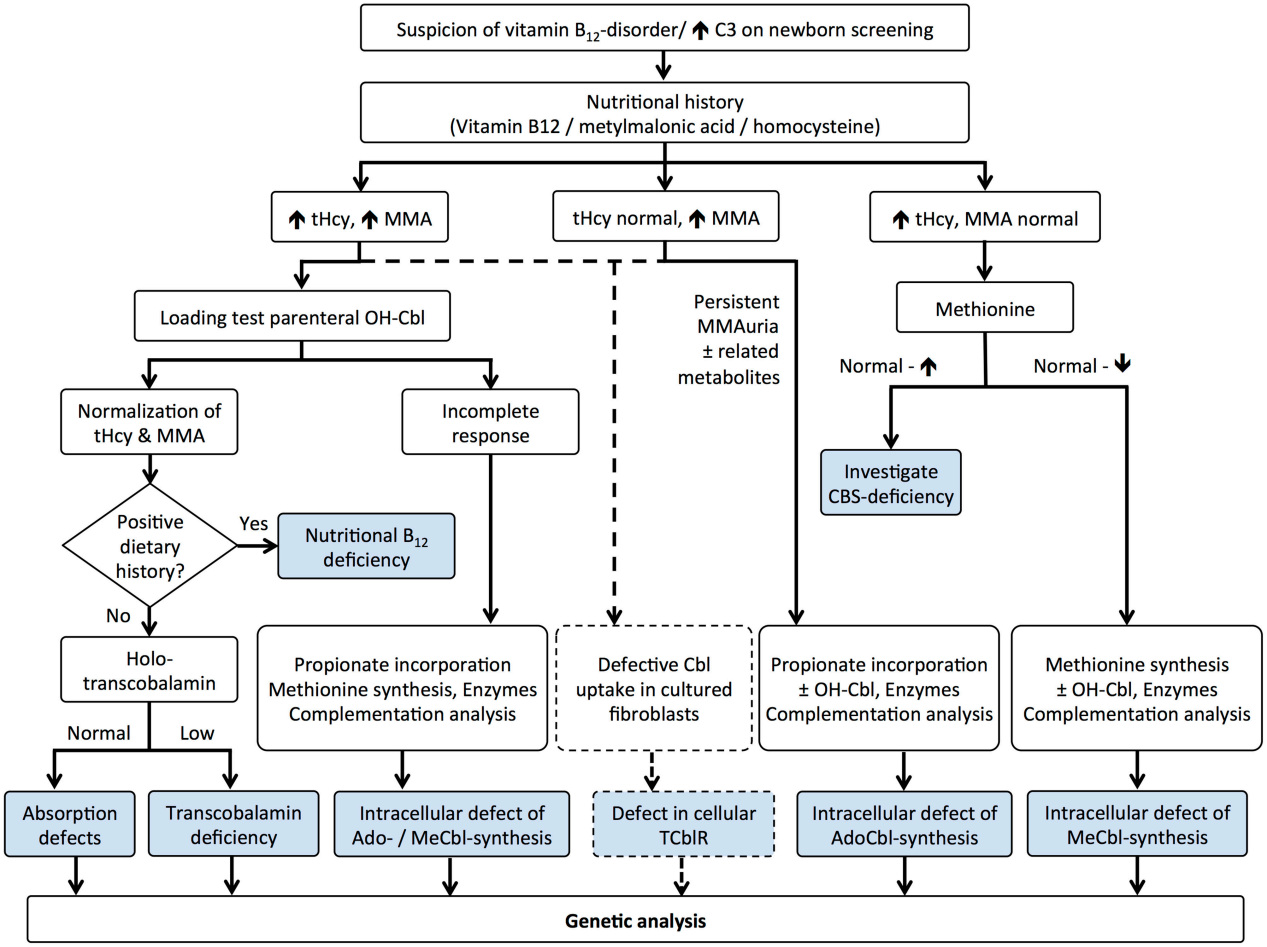
**Algorithm for the diagnosis of vitamin B_12_ deficiency in neonates: metabolomic and functional studies**. Elevated propionylcarnitine (C3) prompts the analysis of nutritional history with examination of vitamin B_12_ and its metabolites, tHcy, and MMA. Combined hyperhomocysteinemia and methylmalonic acidemia may be caused by insufficient B_12_ intake, defective absorption (intrinsic factor, transcobalamin) and genetic mutations affecting uptake (transcobalamin receptor, TCblR), lysosomal exiting (cblF and cblJ), processing (cblC), and trafficking (cblD). Total or incomplete response to a high dose of HOCbl helps to elucidate the cause of the dysfunction. Importantly, mutations in the TCblR gene may present with either the combined phenotype or with isolated methylmalonic acidemia, which should be considered using flow charts (dotted lines). Classical isolated hyperhomocysteinemia and methylmalonic acidemia are typically confirmed by functional assays, except for CBS deficiency that presents with normal to high methionine levels. Mutations affecting methionine synthase (cblG) and its partner reductase (cblE) lead to isolated hyperhomocysteinemia, whereas mutations affecting MCM (mut) and its associated proteins (cblA, cblB) result in isolated methylmalonic acidemia. Final confirmation of the disease is carried out by gene sequencing. This figure was modified from Baumgartner and Fowler (2014).

A number of studies point to the lack of correlation between serum and cellular levels of vitamin B_12_ (Carmel, 2000; Solomon, 2005; Devalia et al., 2014; Lysne et al., 2016), and this is particularly important in the case of inborn errors of cobalamin metabolism whereby serum levels of the micronutrient are within the normal range(Watkins and Rosenblatt, 2013). In this regard, serum vitamin B_12_ and holo-TC should be avoided as sole markers of B_12_ deficiency in neonatal screening. Time-consuming metabolic studies to uncover genetic complementation or lack of function continue to be utilized in a very limited number of metabolic centers worldwide (Figure 3). In newborn screenings where biochemical markers are indicative of vitamin B_12_ deficiency, the diagnosis of inborn errors of vitamin B_12_ metabolism is performed via functional studies on cultured fibroblasts isolated from skin biopsies (Watkins and Rosenblatt, 2013) or via molecular genetic analysis of putative genes. Functional studies include [^14^C]-propionate or [^14^C]-*N*^5−^methyl-tetrahydrofolate incorporation into cellular macromolecules, to assess the activities of MCM and MS, respectively, and biosynthesis of MeCbl and AdoCbl upon metabolic labeling with holo-TC made from apo-TC and commercially available [^57^Co]-vitamin B_12_ (Watkins and Rosenblatt, 2013). Decreased uptake of [^57^Co]-vitamin B_12_ is indicative of impaired receptor-mediated endocytosis of holo-TC by the transcobalamin receptor. Accumulation of [^57^Co]-vitamin B_12_ in the lysosome suggests a dysfunctional cblF or cblJ protein unable to mediate exit of B_12_ from the organelle (Figure 1). Isolated or combined disruption of MeCbl and AdoCbl levels indicates failures in intracellular processing (cblC, cblX), trafficking (cblD), and/or coenzyme biosynthesis and utilization (cblA, cblB, cblG, cblE, mut). When results from [^14^C]-propionate or [^14^C]-*N*^5^-methyl-tetrahydrofolate incorporation suggest abnormal vitamin B_12_ metabolism, somatic cell complementation analysis usually follows (Watkins and Rosenblatt, 2013). Cells from patients are fused with cells from other patients with confirmed mutations using polyethylenglycol and the [^14^C]-propionate or [^14^C]-*N*^5^-methyl-tetrahydrofolate incorporation studies are repeated to interrogate complementation of function. If the incorporation is corrected with respect to control cell lines, then the two cell lines belong to different complementation groups. In contrast, lower than normal [^14^C]-propionate or [^14^C]-*N*^5^-methyl-tetrahydrofolate incorporation after fusion of cell lines with polyethylenglycol confirms that the two patients possess the same genetic defect (Watkins and Rosenblatt, 2013). Nowadays, the vast majority of genes whose mutation lead to functional vitamin B_12_ deficiency have been identified, and therefore, sequencing of these genes or next generation sequencing permits unequivocal diagnosis (Pupavac et al., 2016).

### Combined B_12_ and iron deficiency

Iron deficiency is a condition that could lead to masking of megaloblastic anemia caused by vitamin B_12_ and folate deficiencies (Remacha et al., 2013). The combined deficiency of iron and vitamin B_12_ deficiency is more prominent in individuals aged 60 and older. In general, albeit not always, iron deficiency leads to microcytosis whereas vitamin B_12_ and folate deficiencies result in macrocytosis (Green, 2012). An algorithm to distinguish between these two large classes of anemia based on a decreased in RBC count, hematocrit and hemoglobin levels has been described (Green, 2012). In addition, an algorithm that introduces age and tHcy levels has been developed to discriminate iron deficiency anemia vs. iron and vitamin B_12_ combined anemia (Remacha et al., 2013).

## Vitamin B_12_ status in special populations

### The elderly

Despite an apparent sufficient intake, vitamin B_12_ deficiency is common among the elderly, and the prevalence is shown to increase with age (Allen, 2009; Miles et al., 2015). This is most likely not a physiological change due to aging *per se*, but a consequence of the elderly being more prone to gastric dysfunction, with variable degrees of gastric atrophy and achlorhydria interfering with the absorption of vitamin B_12_ from foods (Carmel, 1997). Most cases of low vitamin B_12_ among the elderly are accompanied by metabolic changes related to deficiency (Carmel, 1997). However, there is limited evidence from observational research linking this to clinical symptoms of deficiency (Miles et al., 2015). Because these metabolic changes usually respond to treatment with oral vitamin B_12_, low serum concentrations of vitamin B_12_ in the absence of clinical symptoms should be regarded as a state of subclinical deficiency. An increasing folate status due to folic acid fortification and/or folic acid supplementation has been demonstrated to accelerate and worsen both the metabolic and the clinical consequences of B_12_ deficiency (Morris et al., 2007; Selhub et al., 2007). Low vitamin B_12_ levels among the elderly should be considered grounds for concern in countries with mandatory folic acid fortification.

### Bariatric surgery and gastrointestinal disorders

The absorption of vitamin B_12_ is largely dependent on a healthy gastrointestinal tract, including (i) the production of intrinsic factor by parietal cells in the stomach, (ii) the dissociation of B_12_ from haptocorrin and binding to intrinsic factor in the neutral environment of the duodenum, and (iii) the uptake of vitamin B_12_ in the ileum. Pernicious anemia, an autoimmune disorder against gastric parietal cells, and bariatric surgery put the patient at risk of developing vitamin B_12_ deficiency by limiting intestinal absorption and/or by reduction of food intake. In addition to reducing intake of the vitamin itself, bariatric surgery may diminish the secretion of intrinsic factor in the stomach, which is normally produced and released in response to food intake (Marcuard et al., 1989). Finally, limited contact with the pancreatic juice due to a shorter common intestinal tract after surgery may hinder the release from haptocorrin, limiting the intestinal uptake. High prevalence of B_12_ deficiency has frequently been reported in subjects undergoing bariatric surgery (Sumner et al., 1996; Shah et al., 2006), and low levels of serum cobalamin is reported to be more indicative of true deficiency in bariatric surgery patients compared to healthy controls (Sumner et al., 1996). The malabsorptive procedures have been shown to yield higher risk of B_12_ deficiency compared to the restrictive procedures (Kwon et al., 2014). In studies where the patients received post-operative intramuscular supplementation of vitamin B_12_, however, no increased risk of becoming deficient is observed (Kwon et al., 2014). In a study of vitamin B_12_ status 5 years after surgery, patients who received intramuscular injections of vitamin B_12_ increased their serum concentration, while the concentration among those not receiving injections decreased (Aaseth et al., 2015). Patients who underwent bariatric surgery would benefit from life-long preventive and maintenance supplementation with vitamin B_12_. More broadly, gastrointestinal disorders that affect the small intestine (for example, ileal resection) could increase the risk of B_12_ deficiency through malabsorption (Battat et al., 2014). High prevalence of vitamin B_12_deficiency has been reported among untreated celiac disease patients (Theethira et al., 2014). Hence, patients newly diagnosed with celiac disease should be screened for B_12_ deficiency. Vitamin B_12_ deficiency is also frequently associated with chronic gastritis, which is most often a consequence of *H. pylori* infection (Varbanova et al., 2014).

### Neurological disorders

The consequences of vitamin B_12_ deficiency at the onset and progression of neurological impairments are well established (Kumar, 2014). In contrast, the causative role of vitamin B_12_ deficiency in neurological disorders such as Parkinson's, Alzheimer's, and others is controversial. A review of 43 studies worldwide showed that low serum vitamin B_12_ levels correlate with increased neurodegenerative disease and cognitive impairment (Moore et al., 2012). While a small subset of dementias examined in some of these studies responded favorably to vitamin B_12_ supplementation, no benefit was observed in patients with an established, pre-existing deficiency of vitamin B_12_ (Moore et al., 2012). One explanation may be that at least some of the neurological consequences of vitamin B_12_ deficiency are irreversible. Another possibility is that the relationship between B_12_ status and neurological disorders is not causal. A comprehensive analysis of studies performed from 2002 to 2012 examined tHcy levels with respect to dementia and cognitive decline (Health Quality Ontario, 2013). The results were controversial based on sample size and study design. Although the authors described an association between elevated tHcy and the onset of dementia, treatment with vitamin B_12_ did not improve cognitive function (Health Quality Ontario, 2013). On the other hand, a slower rate of brain atrophy was noted in patients with mild cognitive impairment who have received treatment with vitamin B_12_ (effective with both oral and parenteral administration) and folate (Health Quality Ontario, 2013). Elevated tHcy and MMA were identified in a small cohort of patients with Parkinson's disease, progressive supranuclear palsy and ammyotrophic lateral sclerosis compared to healthy subjects (Levin et al., 2010). In all cases, patients with these neurological diseases presented serum vitamin B_12_ and folate comparable to that of healthy controls(Levin et al., 2010), suggesting a functional vitamin deficiency. It is unclear whether increased tHcy and MMA contribute to the onset and progression of neurological disease and whether supplementation with cobalamin, in spite of normal serum levels of the micronutrient, may help to reduce these neurotoxic metabolites. It has also been suggested that treatment of Parkinson's and other neurological diseases with levodopa results in elevation of tHcy through S-adenosylmethionine-dependent methylation of levodopa by catechol O-methyl-transferase (Muller, 2009; Muller et al., 2013). The authors proposed that treatment of these diseases with a combination of levodopa and an inhibitor of O-methyl-transferase may prevent tHcy elevation and oxidative stress (Muller, 2009). Advanced-stage patients with Parkinson's disease typically receive continuous intraduodenal infusion of levodopa or carbidopa intestinal gel (Muller et al., 2013). These patients develop peripheral neuropathies thought to arise from transient deficiency of vitamins B_6_ and B_12_ as judged by a favorable response to concomitant supplementation with these micronutrients (Muller et al., 2013). A study performed with 1354 elder Australian subjects showed an association between low vitamin B_12_ levels and cognitive impairments that was enhanced in the presence of high folate intake (Moore et al., 2014). Furthermore, the study identified that subjects exhibiting normal serum vitamin B_12_ levels in the presence of high erythrocyte folate were also more likely to develop cognitive impairment (Moore et al., 2014). Unfortunately, this study did not determine metabolic markers of vitamin B_12_ deficiency such as MMA or tHcy. Altogether, available data suggests that preventing a deficiency of vitamin B_12_ may afford protection from neurological deterioration, and that some but not all cognitive impairments respond to therapy with vitamin B_12_. In terms of causation, the contribution of insufficient vitamin B_12_ metabolism to the onset and progression of unrelated disorders such as Parkinson's and Alzheimer's diseases and other dementias awaits the implementation of large population clinical trials.

### Autoimmune lymphoproliferative syndrome

ALPS is a genetic disorder leading to defective lymphocyte apoptosis (Oliveira, 2013; Shah et al., 2014). This results in an accumulation of lymphocytes, splenomegaly, multilineage cytopenias, lymphadenopathy, hepatomegaly, and an increased risk of B-cell lymphoma. The disease is accompanied nearly always by autoimmune manifestations (Jackson et al., 1999; Oliveira et al., 2010). Most ALPS patients are affected by heterozygous germline mutations in the Fas cell surface death receptor gene (*FAS*) via autosomal dominant inheritance (Jackson et al., 1999; Holzelova et al., 2004; Oliveira et al., 2010). Somatic mutations in *FAS* have also been described albeit less frequently (Jackson et al., 1999; Holzelova et al., 2004; Oliveira et al., 2010). FAS is a member of the tumor necrosis factor receptor family (FAS/CD95/APO-1/TNFRSF6) and it is highly expressed in activated B and T cells (Nagata and Golstein, 1995). FAS-FAS ligand recognition in activated T lymphocytes leads to the activation of the caspase cascade and cellular apoptosis (Nagata and Golstein, 1995). This apoptotic pathway is important for the down-regulation of the immune response, and its disturbance leads to buildup of proliferating lymphocytes resulting in lymphoid hyperplasia and autoimmunity (Rieux-Laucat et al., 2003). Revised diagnostic criteria and classification for the ALPS syndrome were established in 2009 at a US National Institutes of Health International Workshop. Required criteria for ALPS included the presence of lymphadenopathy and/or splenomegaly and elevated TCRαß^+^-DNT cells. Accessory criteria for ALPS were subdivided into primary (abnormal lymphocyte apoptosis assay and presence of pathogenic mutations in genes of the FAS pathway) and secondary criteria, which included characteristic histopathology, the combined presence of autoimmune cytopenia, polyclonal hypergamma-globulinemia, an ALPS-compatible family history, and the presence of elevated biomarkers, such as interleukin-10, interleukin-18, soluble FAS ligand, and vitamin B_12_ (Oliveira et al., 2010). These biomarkers are included in the diagnostic criteria because they can predict both germline and somatic FAS mutations with a post-test probability from 85 to 97% (87% for vitamin B_12_) and because they are accessible to facilities without the ability to do genetic analysis or functional assays (Teachey et al., 2005; Magerus-Chatinet et al., 2009; Caminha et al., 2010; Seif et al., 2010). Very highly elevated median serum vitamin B_12_ in ALPS Ia and Ia-s (1667 pmol/L; 1220 pmol/L) compared with mutation-negative relatives (350 pmol/L; *P* < 0.0001) and healthy mutation-positive relatives (421 pmol/L; *P* < 0.0001) have been reported (Caminha et al., 2010). In particular, serum vitamin B_12_ median levels in ALPS III and ALPS-Phenotype were substantially elevated (560 and 696 pmol/L) (Caminha et al., 2010). Another group observed significantly higher mean serum vitamin B_12_ concentrations (3900 pmol/L; controls: 274 pmol/L; *p* < 0.0001) and significantly higher mean holo-haptocorrin (holo-HC) concentrations (3810 pmol/L; controls: 194 pmol/L; *p* < 0.0001) in the presence of normal holo-TC concentrations (Bowen et al., 2012). The abnormally high levels of circulating vitamin B_12_ found in ALPS patients has been ascribed to high expression of HC by lymphocytes isolated from ALPS patients, a characteristic not observed in lymphocytes isolated from healthy control subjects (Bowen et al., 2012). Thus, it would appear that HC expression is upregulated in ALPS. In the Bowen et al. study, the metabolic markers of vitamin B_12_ status (tHcy and MMA) in ALPS patients were investigated and no differences with respect to healthy controls were found (Bowen et al., 2012). This is consistent with the fact that high serum holo-HC does not impair holo-TC uptake by the cellular TCblR (Quadros et al., 2009, 2010; Jiang et al., 2013; Quadros and Sequeira, 2013) and the downstream intracellular utilization of the micronutrient (Hannibal et al., 2009, 2013). It remains to be determined whether excess serum vitamin B_12_ found in ALPS patients derives from clearance of liver storages or simply by increased dietary assimilation.

### Vegetarians and vegans

Although a properly planned vegetarian diet is regarded as nutritionally adequate and healthy, unsupplemented populations who actively avoid food of animal origin are at high risk of insufficient dietary intake of vitamin B_12_. The amount of B_12_ in vegetarian diets depends on the extent of inclusion of animal products or the use of dietary supplements. Supplementation is recommended to those completely avoiding animal products (strict vegans). Compared to meat eaters, both vegetarians and particularly vegans have a lower intake of vitamin B_12_ (Davey et al., 2003; Rizzo et al., 2013; Kristensen et al., 2015; Schupbach et al., 2015). This is also reflected by lower serum concentrations (Gilsing et al., 2010), and vitamin B_12_ deficiency is shown to be common following all types of vegetarian diets (Pawlak et al., 2013, 2014). However, in a study comparing micronutrient status in omnivores, vegetarians and vegans from Switzerland, a low vitamin B_12_ intake among vegans was not accompanied by low serum concentrations of the micronutrient (Schupbach et al., 2015). This was attributed to widespread use of B_12_ supplements, which adheres to dietary recommendations for this subpopulation (Pawlak et al., 2014). Infants of vegan mothers not taking supplements are also at increased risk of becoming vitamin B_12_ deficient, especially so when exclusively breastfed (Dror and Allen, 2008). This is due both to small hepatic stores and low intake through the breast milk, both a result of poor maternal vitamin B_12_ status. In light of these findings, it is advised that vegetarians and vegans monitor their serum vitamin B_12_ levels and at least one metabolic marker (tHcy or MMA), or consider taking a B_12_ supplement as part of their dietary plan.

### Medication affecting vitamin B_12_ status

Besides factors influencing the intake and absorption of vitamin B_12_, some medications have been shown to interfere with vitamin B_12_ absorption or metabolism, potentially putting certain patient groups at risk of developing vitamin B_12_deficiency. Metformin (Glucophage) is regarded a first-line drug in the treatment of type 2 diabetes mellitus. However, it has been shown that treatment with metformin is associated with a decrease in serum vitamin B_12_ concentrations, which worsens with increasing dose and length of treatment (Ting et al., 2006; de Jager et al., 2010; Liu et al., 2014). The association has been suggested to be due to interference with the calcium dependent intestinal uptake of the vitamin B_12_-intrinsic factor complex (Bauman et al., 2000). However, although serum B_12_ is reduced, metformin treatment has been associated with reduction in plasma MMA and indications of improved intracellular B_12_ status in diabetics (Obeid et al., 2013). Hence, a vitamin B_12_ resistance phenomenon was suggested in diabetics, and metformin treatment seemed to improve the intracellular vitamin B_12_ metabolism, contrary to previous beliefs. Two studies found that patients treated with metformin had reduced levels of both serum vitamin B_12_ and haptocorrin, but treatment was without effect on the levels of holo-TC, the bioactive form of vitamin B_12_ that is distributed systemically to all cells in the body (Leung et al., 2010; Greibe et al., 2013c). Another study showed that treatment of rats with metformin increased hepatic accumulation of vitamin B_12_ (Greibe et al., 2013b). This suggests that metformin may alter the homeostasis and tissue distribution of vitamin B_12_, the consequences of which remain to be investigated. Because not all cases of low serum vitamin B_12_ imply deficiency, and normal and high serum levels of vitamin B_12_ are likewise observed in patients with severe functional vitamin B_12_ deficiency, concern has been raised regarding the interpretation of the effects of metformin on vitamin B_12_ status (Obeid, 2014). In terms of prevention, diabetics receiving metformin should be regarded at increased risk, and routine screening and supplementation if necessary, should be recommended (Valdes-Ramos et al., 2015).

Proton pump inhibitors or other medications that suppress acid production in the stomach are frequently associated with reduced serum vitamin B_12_ concentrations (Abraham, 2012). This is due to the acidic environment in the stomach being essential for the release of protein-bound vitamin B_12_, as well as initial binding to haptocorrin (Stabler, 2012). While serum vitamin B_12_ is reduced, it usually stays within the normal range (Sheen and Triadafilopoulos, 2011). Hence, the risk of developing overt deficiency due to use of proton pump inhibitors may be low, but it has been suggested that the risk is higher among elderly and malnourished patients receiving long-term treatment (Sheen and Triadafilopoulos, 2011).

Nitrous oxide, an inhalant used for surgical and dental anesthesia and also as a recreational drug (van Amsterdam et al., 2015), directly affects vitamin B_12_ metabolism by irreversibly oxidizing the cobalt atom in B_12_-dependent MS (Guttormsen et al., 1994; Torri, 2010). When subjects with borderline vitamin B_12_ deficiency are treated with nitrous oxide, rapid onset of neurological symptoms has been reported post treatment (Singer et al., 2008). Patients who are candidates for interventions with nitrous oxide anesthesia may benefit from pre-operatory supplementation with vitamin B_12_.

## Genetic determinants of vitamin B_12_ status

Aside from inborn errors of metabolism affecting the intracellular pathways of vitamin B_12_ trafficking and assimilation (for excellent reviews on the topic see Froese and Gravel, 2010; Watkins and Rosenblatt, 2011), mutations in the transcobalamin gene (TCN2) (Keller et al., 2016), high levels of the soluble transcobalamin receptor (sCD320) Hoffmann-Lucke et al., 2013, and polymorphisms in 3-hydroxybutyryl-CoA hydrolase (HIBCH) (Molloy et al., 2016) also affect the serum markers of vitamin B_12_ status. Mutations in the TCN2 gene can lead to false low levels of circulating holo-TC without effect on other biomarkers of vitamin B_12_ status (Keller et al., 2016). Increased levels of a heavier variant of the sCD320 led to elevated holo-TC and vitamin B_12_ in serum, though the reasons for such variations are currently unknown (Hoffmann-Lucke et al., 2013). The larger apparent molecular weight of sCD320 could be due to altered glycation or formation of higher order oligomers of the sCD320 (Hoffmann-Lucke et al., 2013). A genome-wide analysis in 2210 healthy Irish adults identified strong associations between plasma MMA with SNPs in acylCo-A synthetase (ACSF3) and HIBCH, with these loci accounting for 12% of the variance in MMA concentration (Molloy et al., 2016). HIBCH catalyzes a unique step in the degradation of valine, converting 3-hydroxyisobutyric acid-CoA to 3-hydroxyisobutyric acid. The presence of polymorphisms in HIBCH represents an independent determinant of serum MMA concentrations. The molecular mechanism by which polymorphic variants of HIBCH lead to elevated MMA remain to be investigated (Molloy et al., 2016). Transient elevation of MMA has also been documented in patients with mutations in methylmalonate semialdehyde dehydrogenase (Marcadier et al., 2013), and in ACSF3, which possesses malonyl-CoA and methylmalonyl-CoA activity (Alfares et al., 2011; Sloan et al., 2011). Mutations in mitochondrial succinate-CoA ligase (SUCLG1) also lead to elevated MMA (Valayannopoulos et al., 2010).

Mutations that impair the endocytic and lysosomal pathways independently of vitamin B_12_ metabolism also lead to transient deficiency of the micronutrient, with elevation of tHcy and MMA (Stockler et al., 2014; Dutchak et al., 2015; Zhao et al., 2015). These include abnormal lysosome acidification in a patient with Alzheimer's disease (Zhao et al., 2015), impaired endocytosis due to a mutation in the rabenosyn-5 gene (Stockler et al., 2014; Zhao et al., 2015), and disrupted lysosomal acidification and gene expression in a knock-out mouse model of mTOR inhibitor NPRL2 (Dutchak et al., 2015). This pathway is thought to cause a defective processing of holo-TC in the lysosome (Dutchak et al., 2015).

Upregulation of the expression of hepatic peroxisome proliferator activated receptor alpha (PPAR alpha), has been shown to elevate plasma MMA in male Wistar rats fed a normal high fat diet in the presence of PPAR alpha agonist tetradecylthioacetic acid (TTA) (Lysne et al., 2016). Because the amount of receptor is a limiting factor for PPAR alpha activity, and the amount of PPAR alpha protein strongly correlates with its level of mRNA expression (Lemberger et al., 1996), differential expression of PPAR alpha induced by diet, stress and other factors may represent another vitamin B_12_-independent determinant of plasma MMA.

Overall, the finding that mutations and polymorphisms in genes peripheral or unrelated to intracellular vitamin B_12_ pathways affect its marker metabolites, substantiates the need for the combined analysis of at least two biomarkers for the accurate assessment of vitamin B_12_ status.

## Concluding remarks

The diagnosis of vitamin B_12_ deficiency both in children and adults requires the use of at least two biomarkers. Among the biomarkers currently available for diagnostic purposes, total serum vitamin B_12_ is equivocal for the identification of functional deficiencies, such as those caused by inborn errors of metabolism. The use of holo-TC to determine vitamin B_12_ status is advantageous in that it reflects the biologically active pool of vitamin B_12_ in serum, but its homeostasis is poorly understood. This is illustrated by the abnormal levels of holo-TC observed under various unrelated disease states and in individuals possessing otherwise normal total serum vitamin B_12_. tHcy is particularly useful as a marker of vitamin B_12_ status in the neonate, but its reliability and specificity are equivocal in adulthood because it is influenced by many factors such as folate status. Methylmalonic acid continues to be the most sensitive and specific marker for vitamin B_12_ status in individuals of all ages with normal renal function. The use of acylcarnitines can be considered ancillary to diagnosis, but certainly not a first-line test. One major challenge lies in identifying early markers of low vitamin B_12_ status for the timely diagnosis of the clinically and biochemically silent subclinical vitamin B_12_ deficiency. Likewise, the consequences of insufficient vitamin B_12_ levels in special populations such as the elderly, vegetarians and vegans, infants, and pregnant women suffer from poor awareness by both patients and clinicians. The finding that low vitamin B_12_ status is associated with more prominent metabolic markers of vitamin B_12_ deficiency in the presence of high folic acid concentrations (Miller et al., 2009) points to the importance of nutrient-nutrient interactions, which have only been considered over the past decade and is of particular relevance to countries with ongoing folic acid fortification of foods.

## Author contributions

All authors listed, have made substantial, direct and intellectual contribution to the work, and approved it for publication.

### Conflict of interest statement

The authors declare that the research was conducted in the absence of any commercial or financial relationships that could be construed as a potential conflict of interest.
