# Corrigendum: Biomarkers and Algorithms for the Diagnosis of Vitamin B_12_ Deficiency

**DOI:** 10.3389/fmolb.2017.00053

**Published:** 2017-08-08

**Authors:** Luciana Hannibal, Vegard Lysne, Anne-Lise Bjørke-Monsen, Sidney Behringer, Sarah C. Grünert, Ute Spiekerkoetter, Donald W. Jacobsen, Henk J. Blom

**Affiliations:** ^1^Laboratory of Clinical Biochemistry and Metabolism, Department for Pediatrics, Medical Center, University of Freiburg Freiburg, Germany; ^2^Department of Clinical Sciences, University of Bergen Bergen, Norway; ^3^Department of Clinical Biochemistry, Haukeland University Hospital Bergen, Norway; ^4^Department of Cellular and Molecular Medicine, Lerner Research Institute, Cleveland Clinic Cleveland, OH, United States

**Keywords:** vitamin B_12_, cobalamin, homocysteine, methylmalonic acid, holo-transcobalamin, diagnostic algorithm, functional deficiency of B_12_

There was an error in one sentence citing work by (Moore et al., 2012): “While a small subset of dementias examined in some of these studies responded favorably to vitamin B_12_ supplementation, no benefit was observed in patients with an established, pre-existing deficiency of vitamin B_12_ (Moore et al., 2012).”

The correct sentence is: “While a small subset of dementias examined in some of these studies responded favorably to vitamin B_12_ supplementation, benefit was only observed in patients with an established, pre-existing deficiency of vitamin B_12_ (Moore et al., 2012).” The authors apologize for the mistake. This error does not change the scientific conclusions of the article in any way.

## Conflict of interest statement

The authors declare that the research was conducted in the absence of any commercial or financial relationships that could be construed as a potential conflict of interest.
